# Supplementary material for: Unsupervised machine learning reveals key immune cell subsets in COVID-19, rhinovirus infection, and cancer therapy
Source: eLife. 2021 Aug 5;10:e64653. doi: 10.7554/eLife.64653 (PMC8370768; doi:10.7554/eLife.64653)
Supplement: Transparent reporting form [file elife-64653-transrepform1.pdf]

eLIFE
Westbrook Centre, Milton Road
Cambridge CB4 1YG
UK
P 01223 855340
W elifesciences.org
T @elife

eLife's transparent reporting form

We encourage authors to provide detailed information *within their submission* to facilitate the interpretation and replication of experiments. Authors can upload supporting documentation to indicate the use of appropriate reporting guidelines for health-related research (see EQUATOR Network), life science research (see the BioSharing Information Resource), or the ARRIVE guidelines for reporting work involving animal research. Where applicable, authors should refer to any relevant reporting standards documents in this form.

If you have any questions, please consult our Journal Policies and/or contact us: editorial@elifesciences.org.

Sample-size estimation

- You should state whether an appropriate sample size was computed when the study was being designed
- You should state the statistical method of sample size computation and any required assumptions
- If no explicit power analysis was used, you should describe how you decided what sample (replicate) size (number) to use

Please outline where this information can be found within the submission (e.g., sections or figure legends), or explain why this information doesn't apply to your submission:

This study considered 106 samples from 35 different individual donors spanning four datasets (Supplemental Table 1) and including 10s of millions of cells. This included 32 samples of blood from rhinovirus subjects (4 timepoints from n = 8 individuals, new data) analyzed by spectral flow cytometry. Data from published work included 40 samples of blood from melanoma patients (4 timepoints from n = 10 individuals, Greenplate et al. *Cancer Immunol Res* 2019), 24 samples of blood from COVID-19 patients (2 samples from n = 12 individuals, Rodriguez et al., *bioRxiv* & *Cell Reports Medicine* 2020), and 10 samples of blood from acute myeloid leukemia patients (2 timepoints from n = 5 individuals, Ferrell et al., *PLoS One* 2016); all of these are mass cytometry data, are cited in the manuscript, and are available online. For the purposes of this study, previously published works respectively had sample sizes of paired data per patient ranging from 5 to 12 patient samples and the new study included 8 individuals. Another aspect is the number of cells per sample. For the new rhinovirus datasets, cells analyzed typically were millions of individual CD4+ T cells. For the published datasets, cells analyzed were on the order of 10s to 100s of thousands of individual cells, as mass cytometry typically collects fewer cells per sample. The algorithm uses matched pairs of samples from individuals and does not work with a single sample from one donor. The goal of the study was to validate the analysis method using as many samples as provided in previous work and as collected in the spectral flow cytometry experiment (i.e., all usable pairs of samples meeting study criteria were included).

eLife Sciences Publications, Ltd is a limited liability non-profit non-stock corporation incorporated in the State of Delaware, USA, with company number 5030732, and is registered in the UK with company number FC030576 and branch number BR015634 at the address Westbrook Centre, Milton Road Cambridge CB4 1YG, UK | March 2019

Replicates

- You should report how often each experiment was performed
- You should include a definition of biological versus technical replication
- The data obtained should be provided and sufficient information should be provided to indicate the number of independent biological and/or technical replicates
- If you encountered any outliers, you should describe how these were handled
- Criteria for exclusion/inclusion of data should be clearly stated
- High-throughput sequence data should be uploaded before submission, with a private link for reviewers provided (these are available from both GEO and ArrayExpress)

Please outline where this information can be found within the submission (e.g., sections or figure legends), or explain why this information doesn't apply to your submission:

Spectral flow cytometry experiments were performed once on each patient sample. Data were included from previously published works if there were paired samples per individual patient or study subject (Figure 6). Inclusion and exclusion of data is further described in Methods under "Data pre-processing." T-REX was tested with paired samples from individuals infected with rhinovirus (Dataset 1, n = 8 pairs), patients with moderate or severe COVID-19 (Dataset 2, n = 12 pairs, Rodriguez et al., Cell Reports Medicine 2020), melanoma patients being treated with α-PD-1 checkpoint inhibitor therapies (Dataset 3, n = 5 pairs, Greenplate et al., *Cancer Immunol Res* 2019), and acute myeloid leukemia patients undergoing induction chemotherapy (Dataset 4, n = 10 pairs, Ferrell et al., *PLoS One* 2016).

Statistical reporting

- Statistical analysis methods should be described and justified
- Raw data should be presented in figures whenever informative to do so (typically when N per group is less than 10)
- For each experiment, you should identify the statistical tests used, exact values of N, definitions of center, methods of multiple test correction, and dispersion and precision measures (e.g., mean, median, SD, SEM, confidence intervals; and, for the major substantive results, a measure of effect size (e.g., Pearson's r, Cohen's d)
- Report exact p-values wherever possible alongside the summary statistics and 95% confidence intervals. These should be reported for all key questions and not only when the p-value is less than 0.05.

Please outline where this information can be found within the submission (e.g., sections or figure legends), or explain why this information doesn't apply to your submission:

One of the main goals of this paper was to validate a newly developed method of analysis using a range of translational disease settings (viral infection and two contrasting cancer therapies); therefore, to test the approach, T-REX was applied to paired samples from previously published works in addition to a newly generated dataset (Figure 6). Details about statistical cutoffs used in T-REX are provided in Results under "T-REX identifies cells in phenotypically distinct regions of significant change" (Figure 2). Iterative testing of *k*-values is in Results under "A k-value of 60 effectively identified immune hotspots in T-REX" (Figure 4). Additional information about median, IQR, mean, SD, p-values, and other key quantitative decisions is reported and labeled throughout the Results section and in figure legends.

(For large datasets, or papers with a very large number of statistical tests, you may upload a single table file with tests, Ns, etc., with reference to sections in the manuscript.)

Group allocation

- Indicate how samples were allocated into experimental groups (in the case of clinical studies, please specify allocation to treatment method); if randomization was used, please also state if restricted randomization was applied
- Indicate if masking was used during group allocation, data collection and/or data analysis

Please outline where this information can be found within the submission (e.g., sections or figure legends), or explain why this information doesn't apply to your submission:

T-REX is an unsupervised analysis method and was designed to operate using statistical rules that do not take into account any knowledge of classifications. Thus, group allocation or outcome data were not used in analyzing the data and the outcome groupings were used once the T-REX algorithm already found regions of change. For this study, the key external classification of interest was whether cells were virus-specific, which is tracked using tetramers that were not used during the analysis. In addition to this focal question of identifying virus-specific cells, some readers may be interested in additional characteristics of the patient cohorts, such as disease severity or response to treatment. Notably, the focus of this study was NOT to compare such clinical outcomes, and the study was powered to focus on cells by looking at millions of cells per sample and not powered to look at trends across patients. However, future meta-analysis may be able to include the work here, and so we have reported the clinical information available for individuals studied here. For the rhinovirus study, subjects were judged to be infected if they seroconverted to the challenge virus by study day 28 (≥4-fold increase in titer) and/or shed virus in nasal wash specimens during the first 5 days of infection, according to standard protocols. For all other studies, outcome data were provided in the previous publications (Ferrell et al., PLoS One 2016, Greenplate et al. Cancer Immunol Res 2019, Rodriguez et al., medRxiv, Cell Reports Medicine 2020, all of which are cited in the manuscript).

Additional data files ("source data")

- We encourage you to upload relevant additional data files, such as numerical data that are represented as a graph in a figure, or as a summary table
- Where provided, these should be in the most useful format, and they can be uploaded as "Source data" files linked to a main figure or table
- Include model definition files including the full list of parameters used
- Include code used for data analysis (e.g., R, MatLab)
- Avoid stating that data files are "available upon request"

Please indicate the figures or tables for which source data files have been provided:

Numerical data for regions of change are provided in Supplemental Tables 1, 2a, and 2b. Datasets analyzed in this manuscript are available online, including at FlowRepository. COVID-19 Dataset 2 (https://ki.app.box.com/s/sby0jesyu23a65cbgv51vpbzqjdmipr1), melanoma Dataset 3 (http://flowrepository.org/id/FR-FCM-ZYDG), and AML Dataset 4 (http://flowrepository.org/id/FR-FCM-ZZMC) were described and shared online in the associated manuscripts. Rhinovirus Dataset 1 is a newly generated dataset created at the University of Virginia available on FlowRepository (FR-FCM-Z2VX available at: http://flowrepository.org/id/RvFr2DwkDSym1BjCwd7ZJGNbl1ksyXT375C65F282JCjN30meLzqwn8G096D7H9D). Transparent analysis scripts for all four datasets and all presented results are publicly available on the CytoLab Github page for T-REX (https://github.com/cytolab/T-REX) and include open source code and commented Rmarkdown analysis walkthroughs.
